# Association of restless legs syndrome and mortality in end-stage renal disease: an analysis of the United States Renal Data System (USRDS)

**DOI:** 10.1186/s12882-017-0660-0

**Published:** 2017-08-01

**Authors:** Joseph J. DeFerio, Usha Govindarajulu, Amarpali Brar, Daniel Cukor, Kathleen G. Lee, Moro O. Salifu

**Affiliations:** 1 Division of Nephrology, Department of Medicine, SUNY Downstate Medical Center, 450 Clarkson Ave, Box 52, Brooklyn, NY 11203 USA; 2 Department of Epidemiology and Biostatistics, School of Public Health, SUNY Downstate Medical Center, Brooklyn, NY USA; 3 Department of Psychiatry and Behavioral Science, SUNY Downstate Medical Center, Brooklyn, NY USA; 4 Department of Healthcare Policy and Research, Weill Cornell Medicine, Cornell University, New York, NY USA

**Keywords:** Restless legs syndrome, Hemodialysis, End-stage renal disease, Mortality, Diagnosis, USRDS

## Abstract

**Background:**

Objective of the study is to assess prevalence and survival among end stage renal disease patients with restless legs syndrome (RLS) within a national database (USRDS).

**Methods:**

A case-control, retrospective analysis was performed. Differences in characteristics between the groups, RLS and those with no sleep disorder (NSD), were determined using χ2 tests. Cox proportional hazard regression was used to assess survival between those with RLS and propensity score matched controls.

**Results:**

Cases of restless legs syndrome were defined as patients that had received an ICD-9 code of 333.94 at any point during their treatment (*n* = 372). RLS group demonstrated a significantly higher proportion of patients with major depressive disorder, dysthymic disorder, anxiety, depression, minor depressive disorder, and psychological disorder. The difference between the survival was not statistically significant in those without sleep disorder as compared to those with RLS (HR =1.16±0.14, *p* = 0.3).

**Conclusions:**

True prevalence of RLS in dialysis patients can only be estimated if knowledge gap for care providers in diagnosis of RLS is addressed. RLS patients also have increased incidence of certain psychological disorders which needs to be addressed.

**Electronic supplementary material:**

The online version of this article (doi:10.1186/s12882-017-0660-0) contains supplementary material, which is available to authorized users.

## Background

Individuals undergoing hemodialysis therapy due to End-Stage Renal Disease (ESRD) commonly report disturbances in their sleep [1–3]. A review of several studies suggested that the prevalence of sleep symptoms could be as high as 44% [1]. In this population, sleep disorders most often manifest as obstructive sleep apnea, excessive sleepiness, and restless legs syndrome (RLS)/Willis-Ekbom disease [4–6]. The effects of sleep disorders have been well documented, however it is worth noting that disrupted sleep can significantly impact quality of life, resulting in depression, psychological consequences, and even reduced socioeconomic status, particularly in the ESRD population [7–10]. Additionally, recurrent sleep disruption and sleep disorders have been associated with increased risk of cardiovascular disease, coronary artery disease and hypertension, and may also increase mortality [11–17].

Restless legs syndrome is cited as one of the most common movement disorders in the general population, with reported prevalence of 1.2% to 15% [18, 19]. In the end-stage renal disease population however, specifically those on hemodialysis, the prevalence of RLS ranges from 6.6% to 62% [9, 20–23]. This wide variability in reported prevalence may be driven by a number of factors, such as the heterogeneity of study populations, a previous lack of standardized diagnosis criteria, and a number of other confounders and comorbidities. The ESRD population tends to experience a high incidence of paresthesia, itching, cramps and peripheral neuropathy, which have likely contributed to such a high reported prevalence [24, 25]. To address the issues surrounding diagnosis of restless legs syndrome/Willis-Ekbom disease, the International Restless Legs Syndrome Study Group (IRLSSG) issued improved diagnostic criteria in 2012, after reaching an international and interdisciplinary consensus [24].

Despite the improvements to diagnosis criteria, sleep disorders continue to be under-recognized by renal healthcare providers [26]. Given the implications of frequent sleep disruption due to sleep disorders, specifically RLS, and the overall inconsistent estimates of prevalence in the ESRD population, we conducted a case-control analysis of the United States Renal Data System (USRDS). Our aim was to clarify RLS diagnosis using *International Classification of Diseases, Ninth Revision, Clinical Modification* (ICD-9-CM) codes, evaluating significant comorbidities, and mortality rates among ESRD patients with and without RLS, with emphasis on those undergoing hemodialysis therapy.

## Methods

De-identified USRDS Standard Analysis Files were used as the source of data in this analysis. Upon data retrieval, there were 2,138,876 patients in the database, however only 1,456,114 patients had a completed medical evidence report on file, with 62.8% of those on hemodialysis therapy. For the study population, however, we only considered ESRD patients greater than 18 years of age, who had initiated hemodialysis therapy between January 1, 2006 and December 31, 2008, and had a post-initiation survival of ≥3 months (*n* = 279,956). This survival criterion was introduced in order to reduce the odds that mortality was due to other acute causes. Patient survival was calculated as the time (in months) from dialysis initiation until transplant, death, or end of study data. Additionally, patients that had been diagnosed with Parkinson’s disease or secondary parkinsonism were excluded to reduce the likelihood of misdiagnosis of RLS due to pre-existing movement disorders [27]. The comorbidities of interest were identified using ICD-9-CM codes (Additional file 1: Table S1). Therefore, cases of restless legs syndrome were defined as patients that had received an ICD-9-CM code of 333.94 at any point during their treatment (*n* = 372), based on the 2012 classification. As a basis for comparison, patients were only considered for selection as a control if they had never been diagnosed with any sleep disorders (NSD) during the study period. After matching, the total number of cases and controls included in the survival analysis was 1092 (RLS, *n* = 273; NSD, *n* = 819). The analysis was approved by the SUNY Downstate Medical Center Institutional Review Board and the USRDS via data use agreements.

### Statistical analysis

Differences in characteristics between the groups (RLS, NSD) were determined using χ2 tests for associations of categorical variables (%), and two-sided two-sample *t*-tests for differences in means (±SD). We then performed propensity score matching between RLS cases and controls by deriving the propensity score from logistic regression based on all variables in Table 1, and conducted greedy matching through a SAS macro for this purpose [28, 29]. Matching was done in a one-to-one stepwise manner, according to propensity score, where the best match was chosen first, followed by the next best until no further matches could be made. The result was a 1:3, cases to controls, matched population. Propensity scores were used as a means to control for any bias due to heterogeneity and imbalance.

**Table 1:** Characteristics of study population and between-group comparison; USRDS 2006–2008

Variable	Total *n* = 279,956	RLS *n* = 372	NSD *n* = 279,584	*P*
Incidence age, years	62.05 ± 15.39	64.22 ± 15.64	62.04 ± 15.39	0.007
Gender				0.2
Female	43.72 (122,400)	47.3 (176)	43.7 (122,224)	
Male	56.28 (157,556)	52.7 (196)	56.3 (157,360)	
Race				<.0001
White	64.38 (180,239)	86.29 (321)	64.35 (179,918)	
Black	29.93 (83,782)	11.56 (43)	29.95 (83,739)	
Asian	3.32 (9282)	1.34 (5)	3.32 (9277)	
Native American	1.08 (3026)	0.27 (1)	1.08 (3025)	
Other/Unknown	1.3 (3627)	0.54 (2)	1.3 (3625)	
Ethnicity				0.002
Non-Hispanic/Latino	85.68 (239,880)	91.4 (340)	85.68 (239,540)	
Hispanic/Latino	14.32 (40,076)	8.6 (32)	14.32 (40,044)	
Vascular access type				0.0002
AV Fistula	14.61 (40,902)	23.12 (86)	14.6 (40,816)	
Graft	3.95 (11,058)	4.3 (16)	3.95 (11,042)	
Catheter	80.28 (224,739)	71.77 (267)	80.29 (224,472)	
Other/Unknown	1.16 (3257)	0.81 (3)	1.16 (3254)	
Body mass index (kg/m^2^)^f^	28.74 ± 7.71	28.98 ± 7.57	28.74 ± 7.71	0.5
Serum creatinine (mg/dl)^f^	6.58 ± 3.46	6.64 ± 3.74	6.58 ± 3.46	0.8
Serum albumin (g/dl)^f^	3.11 ± 0.71	3.32 ± 0.64	3.11 ± 0.71	<.0001
Anemia^a^	8.37 (23,432)	11.83 (44)	8.37 (23,388)	0.02
Cardiovascular				
Coronary artery disease^b^	21.09 (59,054)	17.2 (64)	21.1 (58,990)	0.07
Congestive heart failure	32.18 (90,088)	26.34 (98)	32.19 (89,990)	0.02
Cerebrovascular disease	9.4 (26,329)	8.06 (30)	9.41 (26,299)	0.4
Peripheral vascular disease	14.04 (39,301)	10.22 (38)	14.04 (39,263)	0.03
Hypertension	84.92 (237,737)	83.6 (311)	84.92 (237,426)	0.5
Other				
Diabetes	52.78 (147,771)	38.98 (145)	52.8 (147,626)	<.0001
Cancer	6.81 (19,065)	5.91 (22)	6.81 (19,043)	0.5
Sleep disorders				
Any sleep disorder	0.13 (372)	100.0 (372)	0 (0)	<.0001
Restless legs syndrome	0.13 (372)	100.0 (372)	0 (0)	<.0001
Obstructive sleep apnea	0.01 (17)	4.57 (17)	0 (0)	<.0001
Psychological disorders				
Major depressive disorder	0.37 (1030)	1.61 (6)	0.37 (1024)	<.0001
Dysthymic disorder	0.26 (727)	2.15 (8)	0.26 (719)	<.0001
Anxiety	0.6 (1691)	4.03 (15)	0.6 (1676)	<.0001
Adjustment disorder	0.03 (81)	0.27 (1)	0.03 (80)	0.1
Depression^c^	0.65 (1829)	4.03 (15)	0.65 (1814)	<.0001
Minor depressive disorder^d^	0.29 (808)	2.42 (9)	0.29 (799)	<.0001
Psychological disorder^e^	1.24 (3459)	8.06 (30)	1.23 (3429)	<.0001
Tobacco dependence	6.39 (17,887)	8.87 (33)	6.39 (17,854)	0.05
Alcohol dependence	1.57 (4384)	0.81 (3)	1.57 (4381)	0.2
Drug dependence	1.55 (4339)	1.08 (4)	1.55 (4335)	0.5

Age and clinical measures are mean ± SD. Categorical data are percentages (counts). No sleep disorder diagnosed (NSD)

^a^Determined by ICD-9-CM codes 280.0–280.9, 285.0, 285.2, 285.8, 285.9

^b^Atherosclerosis, myocardial infarction (*MI*), ischemic heart disease (*IHD*)

^c^major depressive disorder, dysthymic disorder, adjustment disorder

^d^Dysthymic disorder, adjustment disorder

^e^Depression, anxiety

^f^Missing data points

To assess survival, we initially produced an unadjusted Kaplan-Meier (KM) survival plot between the cases and controls using time to death, and employed right censoring. Between-group differences were tested by the log-rank test. We then ran an adjusted analysis with Cox proportional hazard regression, with adjustment for incidence age, sex, race, ethnicity, vascular access type, body mass index, serum albumin level, coronary artery disease, congestive heart failure, cerebrovascular disease, peripheral vascular disease, hypertension, diabetes, cancer, major depressive disorder, dysthymic disorder, anxiety, tobacco dependence, and restless legs syndrome. From this, we derived an adjusted survival plot. The survival analysis model was based on significant variables found in the univariable analyses (major depression, anxiety, incidence age, race, ethnicity, etc.) and several factors relevant to and associated with mortality (cardiovascular disease [30, 31], diabetes [32], cancer, BMI, etc).

## Results

Characteristics of the study population are described in Table 1. Of the 279,956 patients examined, there was a mean incidence age of 62 ± 15.4 years. Within this cohort, 64.4% were White, 29.9% Black, 3.3% Asian, 1.1% Native American, and approximately 1.3% other or unknown races. Most patients were non-Hispanic/Latino (85.7%), and over half were male (56.3%). As previously described, individuals with ESRD often suffer from a multitude of metabolic, cardiovascular, and psychological comorbidities. We found a prevalence of diabetes (52.8%), coronary artery disease (21.1%), congestive heart failure (32.2%) and hypertension (84.9%). Psychological disorders were less common, with the greatest proportion having been diagnosed with anxiety (0.6%) and depression (0.7%). Prevalence of RLS was also estimated in this cohort. Three hundred and seventy-two documented cases of diagnosed restless legs syndrome were found which represents 0.1% of the study population.

### Between-group characteristics

Our primary goal, however, was to examine the differences between RLS cases (*n* = 372) and non-sleep disorder (NSD) controls (*n* = 279,584). Table 1 also describes the between-group associations. When stratified by RLS status, significant differences in age and race were found. The cases and controls initiated dialysis therapy at an average of 64.22 ± 15.64 and 62.04 ± 15.39 years, respectively. There was also a larger proportion of Whites with RLS (86.3%) versus the control group (64.4%). Conversely, our data show only 11.6% of the RLS cases identified as Black/African-American, while this group accounted for nearly 30% of the controls. Serum albumin was statistically higher amongst the cases, while the NSD cohort demonstrated a higher proportion of patients with coronary artery disease, congestive heart failure, peripheral vascular disease and diabetes. Despite the relatively smaller number of patients with psychological conditions, the RLS group demonstrated a significantly higher proportion of patients with major depressive disorder, dysthymic disorder, anxiety, depression, minor depressive disorder, and psychological disorders.

### Survival analysis

**Fig. 1:**
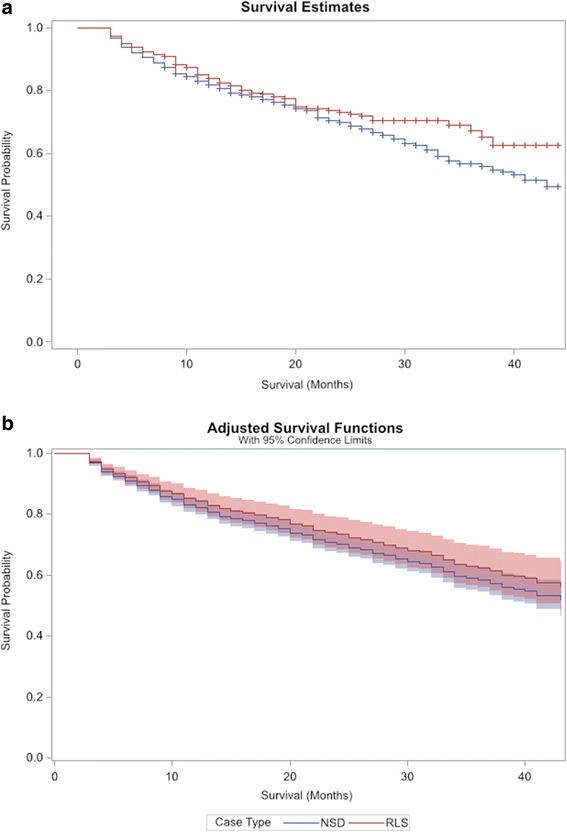
**a** Mortality in patients with ESRD by RLS status, unadjusted crude survival assessed by Kaplan-Meier analysis, Wilcoxon *p* = 0.2. **b** Mortality in patients with ESRD by RLS status, survival assessed by Cox regression analysis including 95% CI (shaded regions)

The Kaplan-Meier plot showed that survival in persons who have RLS was not different than those without RLS (Fig. 1a). The difference between the survival plots was not statistically significant at *p* = 0.2. Using Cox proportional hazards regression (Fig. 1b), the difference between the survival plots was not statistically significant with survival in those without sleep disorder as compared to those with RLS, HR = 1.16 ± 0.14, *p* = 0.3 (Table 2). In univariate analysis, age (*p* < .0001), race (*p* = 0.01), albumin (*p* = 0.0005), congestive heart failure (*p* = 0.004), hypertension (*p* = 0.003), and cancer (*p* = 0.03) were associated with an increase in mortality. However, when these covariates were adjusted for in the Cox models, there was no increased mortality in patients with RLS (Fig. 1).

**Table 2:** Survival analysis – propensity matched population (*n* = 1092)

Variable	β	SE	HR	*P*
Incidence age, years	0.03	0.005	1.03	<.0001
Female	−0.02	0.11	0.98	0.9
Race	0.22	0.09	1.25	0.01
White	−0.53	0.72	0.59	0.5
Black	−0.82	0.73	0.44	0.3
Asian	−1.15	0.79	0.32	0.1
Native American	−1.45	1.02	0.23	0.15
Ethnicity				
Non-Hispanic/Latino	0.26	0.19	1.3	0.2
Vascular access type				
AV Fistula	−0.39	0.42	0.68	0.4
Graft	−0.11	0.46	0.89	0.8
Catheter	−0.07	0.39	0.93	0.9
Body mass index (kg/m^2^)	−0.006	0.009	0.99	0.5
Serum albumin (g/dl)	−0.27	0.08	0.77	0.0005
Cardiovascular				
Coronary artery disease	0.14	0.13	1.15	0.3
Congestive heart failure	0.34	0.12	1.41	0.004
Cerebrovascular disease	0.17	0.17	1.19	0.3
Peripheral vascular disease	0.24	0.15	1.27	0.1
Hypertension	−0.43	0.15	0.65	0.003
Other				
Diabetes	0.1	0.12	1.1	0.4
Cancer	0.39	0.18	1.48	0.03
Psychological disorders				
Major depressive disorder	0.32	0.72	1.37	0.7
Dysthymic disorder	0.63	0.59	1.88	0.4
Anxiety	0.46	0.46	1.57	0.3
Tobacco dependence	0.14	0.22	1.16	0.5
Case type				
No sleep disorders (NSD)	0.15	0.14	1.16	0.3

## Discussion

This study attempted to clarify the associations between RLS diagnosis, patient comorbidities, and mortality within the ESRD patient population, particularly those currently undergoing hemodialysis therapy. It is crucial to understand how sleep disorders like RLS may impact the health outcomes of this population. Results from our analyses reveal two interesting findings: 1) there are no significant differences in mortality between patients who are diagnosed with RLS and those with no sleep disorders, and 2) patients with RLS are more likely to have diagnoses of psychological conditions.

We found a significantly higher proportion of patients with RLS had major depressive disorder, dysthymic disorder, anxiety, depression or minor depressive disorder, as compared to those with no sleep disorder. This may have been related to changes in quality of life in those with RLS. Further, our results did not show increased mortality in patients with RLS as compared to those with no sleep disorders. These findings are similar to Stefanidis et al. who reported no difference in 3-year mortality in 579 hemodialysis patients with and without RLS [33] but are in contrast to other studies which have reported higher mortality in patients with RLS [12, 34]. ESRD studies claiming greater likelihood of mortality for patients with sleep disorders appear to contain relatively homogeneous populations in their analyses. Our study, however, leverages the USRDS which has collected data on ESRD patients undergoing dialysis from across the United States and boasts a very diverse population. In addition, we have used diagnostic codes to assess RLS prevalence and comorbidities, along with cause-of-death reports to determine survival status. The greater heterogeneity in our patient population most likely drives important differences in results between the studies.

In this study, we used ICD-9-CM codes to identify cases of RLS, defined as 1) an urge to move the legs usually but not always accompanied by unpleasant sensations in the legs; 2) the urge to move the legs and unpleasant sensations worsen during periods of rest or inactivity; 3) symptoms are relieved by movement; 4) the patient demonstrates a circadian pattern with peak symptoms occurring at night or during the evening; and 5) the symptoms are distinct from other medical and behavioral conditions. We found a lower prevalence of RLS in the ESRD population than what has been reported in previous studies [12, 35]. One study conducted in India on patients with chronic renal failure also found a very low prevalence (1.5%) [36], however another study conducted by the same group found RLS to be prevalent in 6.6% of patients on hemodialysis [37]. Most previous studies did not use the most recent diagnostic criteria for RLS which could have resulted in improper diagnosis of RLS in those with legs cramps, peripheral neuropathy, positional discomfort, legs swelling, venous stasis, or arthritis. Another distinct difference is that previous studies have been mainly patient-based, conducted in small centers using survey data or diagnostic codes for restless legs syndrome that were lacking [20, 38]. Low reliability of questionnaires as a screening tool for RLS in a population of chronically dialyzed patients seems to be caused by the presence of other legs symptoms in these patients [39].

Lower prevalence of RLS using ICD-9-CM codes may also suggest a gap in knowledge [40] to recognize the disease or reduced priority in terms of documented comorbidities when reporting to Medicare/Medicaid. Kutner et al. found that RLS was considerably underdiagnosed among patients with kidney failure (0.9%) in the USRDS [41]. Unless a patient is proactive or the symptoms are so severe as to be difficult to miss, it is likely that many more cases of RLS are going unrecognized in this population. In fact, the Restless Legs Syndrome Foundation was established to raise awareness of RLS, improve treatments, and through research, find a cure [42, 43]. Although it was formed in 1992, the foundation recognizes that RLS is a common condition which most people do not know about. To address this, the RLS Foundation has established a network of 11 (9 US; 2 Europe) certified Quality Care Centers (QCC) that are staffed by specialists who provide expert care and tailored disease management of RLS. While there is a growing understanding that RLS is more common than previously believed, the QCC network was formed with the idea that as the number of participating hospitals increases, so too will the knowledge of RLS for both patients and physicians. Additionally, it is possible that physicians ascertain RLS to be a symptom of the overall condition of ESRD patients on hemodialysis, rather than a diagnosable condition, and thus fail to register it using ICD-9 coding [44].

Raising awareness and identifying true cases of RLS has greater implications for the health status of ESRD patients on dialysis. Failure to identify and treat this seemingly common condition can add to the stress experienced by ESRD patients and potentially contribute to additional morbidity [41]. Despite a small case population in this study, the proportion of psychological conditions present in the RLS patient group reveals concerning consequences of sleep disorders. This phenomenon, compounded with potential comorbidities found in the population, indicates a need in the medical community to undertake more careful monitoring of mental health status. Of particular concern is that diagnoses of psychological conditions may be under-reported or biased by self-reporting within the population, indicating a greater need for mental health surveillance [45–47]. Whether depression and/or anxiety are contributing to the development of RLS, and vice versa, remains to be determined. However, given the relative treatability of RLS–including iron replacement, drug treatments (dopamine agonists, alpha-2-delta calcium channel ligands, benzodiazepines, etc.), and non-drug therapies (massages, stretching, exercise, applying a cold compress, among others)–it is reasonable to assume that additional screening may improve the health status in this population.

### Limitations

This study has several limitations. Principally, the structure of the USRDS dataset. It is limited by a lack of continuous validation of its methods, lack of complete comorbidity documentation at registration and throughout care, and lack of accuracy of cause-of-death reporting. Data is typically collected via the Centers for Medicare & Medicaid Services (CMS) 2728 form, which is a medical evidence report that is required for all newly diagnosed ESRD patients, regardless of Medicare status or treatment modality. The 2728 form provides critical information to aid caregivers and assure quality of care, however it is not as comprehensive as an electronic health record and is likely limited to the most important or relevant comorbid conditions. The form also describes patient comorbidities as having a current diagnosis or having had the diagnosis in the past ten years, making it difficult to draw strong conclusions about the impact of comorbidity on this patient population. Data is also collected from hospital encounters, however the diagnosis codes are restricted to inpatient visits. This means that physician encounters during dialysis sessions and outpatient visits are not captured, thus restricting the scope of data. The method in which data is collected leaves several variables with missing or incomplete data, and also excludes potentially important co-variables such as parathyroid hormone (PTH) levels, dialysis adequacy, and treatment regimens of RLS.

Therefore, it is reasonable to assume that cases of RLS were missed and might be found in the control group. Similarly, the overall prevalence of anxiety and depression in the population are lower than expected. It has been well documented that ESRD patients on dialysis have a high incidence of depression and anxiety [48–52], however this is not reflected in our dataset. We are unable to determine whether these conditions are typically under-reported by the nephrologists completing the medical evidence forms, by the attending physicians in the hospital, or if the patient population represented by our study do not suffer from mental health and/or sleep disorders at the same rate. Due to the de-identified nature of the USRDS dataset, we are unable to perform sensitivity/specificity analyses to confirm the presence or absence of comorbid conditions.

Finally, contrary to expectations, there was a lack of association between diabetes and mortality, as well as cardiovascular disease and mortality. Given these well-documented associations in previous literature, it is possible that the results suffer from misclassification of exposure biases as we rely solely on inpatient diagnosis data and the 2728 form–of which the limitations have been described.

## Conclusion

This study examined the relationships between RLS diagnosis, comorbidities, and mortality in ESRD patients. Sleep disorders, like RLS, may impact health outcomes of this patient population, particularly those undergoing hemodialysis therapy. We used the USRDS database which has advantages given its size and almost complete inclusion of the ESRD population in the United States, but inherent limitations, such as completeness of data in the Medical Evidence Report at initiation of renal replacement therapy, have been well described.

We conclude that in a nationally representative sample, RLS diagnosis was not associated with mortality in hemodialysis patients. The high prevalence of other psychiatric diagnoses in patients with RLS calls for greater awareness in hemodialysis units but whether screening and management of these psychiatric diagnosis will improve quality of life will need to be tested in prospective studies.

## Additional file

Additional file 1: Table S1. Classification of sleep disorders and associated conditions. (DOCX 17 kb)

## Abbreviations

ESRD: End stage renal disease
ICD-9-CM: International Classification of Diseases-Ninth Revision-Clinical Modification
IRLSSG: International Restless legs Syndrome Study Group
KM: Kaplan Meier
NSD: No sleep disorder
RLS: Restless Legs syndrome
USRDS: United States Renal Data System
