# Supplementary material for: Association of restless legs syndrome and mortality in end-stage renal disease: an analysis of the United States Renal Data System (USRDS)
Source: BMC Nephrol. 2017 Aug 1;18:258. doi: 10.1186/s12882-017-0660-0 (PMC5540277; doi:10.1186/s12882-017-0660-0)
Supplement: Additional file 1: Table S1. — Classification of sleep disorders and associated conditions. (DOCX 17 kb) [file 12882_2017_660_MOESM1_ESM.docx]

| **Additional file 1: Table S1** Classification of Sleep Disorders and Associated Conditions | | | | | | | | | |
| --- | --- | --- | --- | --- | --- | --- | --- | --- | --- |
|  | **ICD-9** | **Description** | | **OSA** | | **RLS** | | **EDS/HPS** |  |
| Sleep Disorders | 291.82 | Alcohol induced sleep disorders |  | |  | | 1 | | |
|  | 292.85 | Drug-induced sleep disorders |  | |  | |  | | |
|  | 307.42 | Persistent disorder of initiating or maintaining sleep |  | |  | |  | | |
|  | 307.45 | Circadian rhythm sleep disorder of nonorganic origin |  | |  | |  | | |
|  | 307.48 | Repetitive intrusions of sleep |  | |  | |  | | |
|  | 327.11 | Idiopathic hypersomnia with long sleep time |  | |  | | 1 | | |
|  | 327.12 | Idiopathic hypersomnia without long sleep time |  | |  | | 1 | | |
|  | 327.20 | Organic sleep apnea, unspecified | 1 | |  | |  | | |
|  | 327.21 | Primary central sleep apnea | 1 | |  | |  | | |
|  | 327.23 | Obstructive sleep apnea | 1 | |  | |  | | |
|  | 327.24 | Idiopathic sleep related non-obstructive alveolar hypoventilation |  | |  | |  | | |
|  | 327.25 | Congenital central alveolar hypoventilation syndrome |  | |  | |  | | |
|  | 327.26 | Sleep related hypoventilation/hypoxemia in conditions classified elsewhere |  | |  | |  | | |
|  | 327.27 | Central sleep apnea in conditions classified elsewhere | 1 | |  | |  | | |
|  | 327.30 | Circadian rhythm sleep disorder; unspecified |  | |  | |  | | |
|  | 327.31 | Circadian rhythm sleep disorder; delayed sleep phase time |  | |  | |  | | |
|  | 327.32 | Circadian rhythm sleep disorder; advanced sleep phase time |  | |  | |  | | |
|  | 327.33 | Circadian rhythm sleep disorder; irregular sleep-wake type |  | |  | |  | | |
|  | 327.36 | Circadian rhythm sleep disorder; shift work type |  | |  | |  | | |
|  | 327.39 | Other circadian rhythm sleep disorder |  | |  | |  | | |
|  | 327.51 | Periodic limb movement |  | |  | |  | | |
|  | 333.94 | Restless leg syndrome |  | | 1 | |  | | |
|  | 347.00 | Narcolepsy, without cataplexy |  | |  | | 1 | | |
|  | 347.01 | Narcolepsy, with cataplexy |  | |  | | 1 | | |
|  | 729.10 | Fibromyalgia |  | |  | |  | | |
|  | 780.09 | Other alterations of consciousness |  | |  | | 1 | | |
|  | 780.51 | Insomnia with sleep apnea, unspecified | 1 | |  | |  | | |
|  | 780.52 | Insomnia, unspecified |  | |  | |  | | |
|  | 780.53 | Hypersomnia with sleep apnea, unspecified | 1 | |  | |  | | |
|  | 780.54 | Hypersomnia, unspecified |  | |  | | 1 | | |
|  | 780.57 | Unspecified sleep apnea | 1 | |  | |  | | |
|  | 786.04 | Cheyne-Stokes respiration |  | |  | |  | | |
|  | 786.09 | Other respiratory abnormalities |  | |  | |  | | |
| Depressive disorders | 296.20 | Major depressive affective disorder, single episode, unspecified |  | |  | |  | | |
|  | 296.21 | Major depressive affective disorder, single episode, mild |  | |  | |  | | |
|  | 296.22 | Major depressive affective disorder, single episode, moderate |  | |  | |  | | |
|  | 296.23 | Major depressive affective disorder, single episode, severe, without mention of psychotic disorder |  | |  | |  | | |
|  | 296.24 | Major depressive affective disorder, single episode, severe, specified as with psychotic disorder |  | |  | |  | | |
|  | 296.25 | Major depressive affective disorder, single episode, in partial or unspecified remission |  | |  | |  | | |
|  | 296.26 | Major depressive affective disorder, single episode, in full remission |  | |  | |  | | |
|  | 296.30 | Major depressive affective disorder, recurrent episode, unspecified |  | |  | |  | | |
|  | 296.31 | Major depressive affective disorder, recurrent episode, mild |  | |  | |  | | |
|  | 296.32 | Major depressive affective disorder, recurrent episode, moderate |  | |  | |  | | |
|  | 296.33 | Major depressive affective disorder, recurrent episode, severe, without mention of psychotic disorder |  | |  | |  | | |
|  | 296.34 | Major depressive affective disorder, recurrent episode, severe, specified as with psychotic disorder |  | |  | |  | | |
|  | 296.35 | Major depressive affective disorder, recurrent episode, in partial or unspecified remission |  | |  | |  | | |
|  | 296.36 | Major depressive affective disorder, recurrent episode, in full remission |  | |  | |  | | |
|  | 300.40 | Dysthymic disorder |  | |  | |  | | |
| Anxiety Disorders | 300.00 | Anxiety state, unspecified |  | |  | |  | | |
|  | 300.01 | Panic disorder without agoraphobia |  | |  | |  | | |
|  | 300.02 | Generalized anxiety disorder |  | |  | |  | | |
|  | 300.09 | Other anxiety states |  | |  | |  | | |
|  | 300.10 | Hysteria, unspecified |  | |  | |  | | |
|  | 300.11 | Conversion disorder |  | |  | |  | | |
|  | 300.20 | Phobia, unspecified |  | |  | |  | | |
|  | 300.21 | Agoraphobia with panic disorder |  | |  | |  | | |
|  | 300.22 | Agoraphobia without mention of panic disorders |  | |  | |  | | |
|  | 300.23 | Social phobia |  | |  | |  | | |
|  | 300.29 | Other isolated or specific phobias |  | |  | |  | | |
|  | 300.30 | Obsessive-compulsive disorder |  | |  | |  | | |
|  | 309.00 | Unspecified nonpsychotic mental disorder |  | |  | |  | | |
| Movement Disorders | 332.00 | Paralysis agitans (Parkinson’s Disease) |  | |  | |  | | |
|  | 332.10 | Secondary parkinsonism |  | |  | |  | | |

OSA: Obstructive sleep apnea; RLS: Restless leg syndrome; EDS/HPS: Excessive daytime sleepiness/hypersomnia
